# Trunk Muscle Endurance, Joint Hypermobility and Postural Alignment in Children with Anorectal Malformation: A Case–Control Study

**DOI:** 10.3390/healthcare14213648

**Published:** 2026-10-28

**Authors:** Zehra Korkut, Hifziye Pervin Nur, Nida Lalecan, Elif Emel Erten, Can Ihsan Oztorun, Seyda Toprak Celenay

**Affiliations:** 1 Department of Physiotherapy and Rehabilitation, Faculty of Health Sciences, Selcuk University, 42250 Konya, Türkiye; 2 Doctorate Program, Institute of Health Sciences, Physiotherapy and Rehabilitation, Ankara Yildirim Beyazit University, 06010 Ankara, Türkiye; pervin.nur99@gmail.com (H.P.N.); nida_lalecan4@hotmail.com (N.L.); 3 Department of Pediatric Surgery, Ankara Bilkent City Hospital, 06800 Ankara, Türkiye; elifemelerten@hotmail.com; 4 Department of Pediatric Surgery, Faculty of Medicine, Ankara Yildirim Beyazit University, 06800 Ankara, Türkiye; drcan-oz@hotmail.com; 5 Department of Physiotherapy and Rehabilitation, Faculty of Health Sciences, Ankara Yildirim Beyazit University, 06760 Ankara, Türkiye; sydtoprak@hotmail.com

**Keywords:** anorectal malformation, core muscle, hypermobility, posture

## Abstract

**Highlights:**

**What are the main findings?**

- Children with anorectal malformations showed lower trunk flexion, extension, and right lateral flexion endurance compared with healthy controls.
- Children with anorectal malformations had higher Beighton Hypermobility Scores and greater postural deviations, particularly in right-sided head, shoulder, and hip alignment.

**What are the implications of the main findings?**

- Assessment of children with anorectal malformations may include musculoskeletal parameters such as trunk muscle endurance, joint hypermobility, and postural alignment.
- When musculoskeletal impairments are identified, individualized rehabilitation strategies addressing both bowel-related symptoms and musculoskeletal dysfunctions may be considered.

**Abstract:**

Background and Objectives: Evaluation of trunk muscle endurance, joint hypermobility and postural alignment in children with anorectal malformation (ARM) may provide useful information for planning comprehensive rehabilitation planning. This study aimed to examine trunk muscle endurance, joint hypermobility and postural alignmentture in children with ARM and to compare these parameters with those of healthy children. Methods: This study included 42 children, comprising children with ARM (ARM group, *n* = 20) and healthy children (control group, *n* = 22). Trunk muscle endurance was assessed with the McGill Trunk Muscle Endurance Tests, joint hypermobility with the Beighton Hypermobility Score (BHS) and postural alignment with the PostureScreen Mobile^®^ application. Independent-samples *t*-tests, chi-square tests, and Benjamini–Hochberg false discovery rate (FDR) correction were used for statistical analysis. Results: Trunk flexion, extension, and right lateral flexion endurance scores were significantly lower in the ARM group than in the control group (*p* < 0.05). In addition, BHS was significantly higher in children with ARM (*p* = 0.008), and the prevalence of joint hypermobility was greater in the ARM group than in the control group. Right head shift, right head tilt angle, right shoulder shift, right hip shift, and left shoulder shift were significantly greater in the ARM group than in the healthy controls (*p* < 0.05). Conclusions: Children with ARM demonstrated reduced trunk muscle endurance, greater joint hypermobility, and selective differences in postural alignment compared with healthy controls. Therefore, assessment of trunk muscle endurance, joint hypermobility, postural alignment, and lumbopelvic function may be considered in children with ARM, with individualized rehabilitation provided when clinically indicated.

## 1. Introduction

Anorectal malformations (ARMs) are congenital anomalies characterized by abnormal development of the anus and rectum, resulting in a range of structural defects [1]. The overall prevalence of ARM has been reported to be approximately 3.32 per 10,000 pregnancies [2]. The literature indicates that ARM may also be accompanied by congenital anomalies, including vertebral, genitourinary, cardiac, and limb anomalies [1,2,3]. Genetic factors, including alterations in the Sonic hedgehog (SHH) signaling pathway, Wnt5a, FGF10, and p63, have also been implicated in the development of ARM [4]. In addition to affecting the gastrointestinal system, ARM may involve the pelvic floor and sphincter complex, as well as the neuromuscular system [5]. These developmental abnormalities in children may lead to long-term functional problems, including defecation disorders, constipation, fecal incontinence, and urinary dysfunction [6]. These problems may affect not only the children’s physical health, but also their daily activities, social participation, and quality of life, thereby imposing a significant psychosocial burden on both children and their families [7].

From a kinetic chain perspective, the body consists of functionally interconnected segments; therefore, structural or functional alterations in one region may influence other components of the chain [8]. Changes in spinal posture and mobility may modify trunk muscle recruitment and lumbopelvic mechanics. These changes may alter the distribution of intra-abdominal pressure and increase the mechanical demands placed on the abdominal and pelvic floor muscles. The literature has indicated that appropriate spinal alignment may facilitate more effective contraction of the pelvic floor and abdominal muscles and may provide better protection against increases in intra-abdominal pressure [9,10]. In addition, some studies have reported negative associations between abdominal muscle endurance and thoracic and lumbar spine curvatures [11]. Therefore, postural differences observed in the spinal and pelvic regions may affect not only muscle function but also the physiological function of the abdominal and pelvic organs [12]. This relationship may also be bidirectional, as bowel dysfunction in children may influence postural development and spinal alignment. In children with ARM, frequently observed chronic defecation problems may lead to repetitive straining and increased intra-abdominal pressure, potentially affecting the function and coordination of the abdominal and pelvic floor muscles and altering trunk stabilization mechanisms. Over time, these changes may increase the demand placed on trunk muscles and contribute to reduced trunk muscle endurance. In one study, it was reported that the pelvic floor muscles of children with ARM were severely underdeveloped and hypoplastic, and that bowel continence was associated with the degree of pelvic floor muscle development [13]. In the pediatric literature, spinal mobility, stability, and lumbopelvic muscle function have been investigated in children with pelvic floor-related conditions such as nocturnal enuresis [10]. In that study, lumbopelvic muscle endurance, stability, and mobility were lower in children and adolescents with nocturnal enuresis than in those without nocturnal enuresis. However, to the best of our knowledge, no previous study has investigated postural alignment or spinal structure in children with ARM.

Joint hypermobility is considered an indicator of connective tissue laxity and may be associated with gastrointestinal, pelvic floor, and urinary system dysfunction in children [14]. De Kort et al. reported that symptoms of non-neurogenic bladder–sphincter dysfunction were more common in children with generalized joint hypermobility. Another study found that children with functional constipation had higher Beighton scores than healthy controls, suggesting a potential association between hypermobility spectrum disorders and constipation and voiding dysfunction [15]. In contrast, Işık et al. found no significant differences in functional constipation or voiding dysfunction between children with and without hypermobility [16]. Given these conflicting findings in the literature, the relationship between generalized joint hypermobility and bowel or bladder dysfunction remains unclear. Moreover, to the best of our knowledge, generalized joint hypermobility has not previously been investigated in children with ARM. Therefore, evaluating hypermobility in this population may help address this gap in the literature.

In this study, postural alignment was operationally defined as two-dimensional linear shift and angular tilt of the head, shoulders, and hips. To the best of our knowledge, no previous study has comprehensively evaluated postural alignment, trunk muscle endurance, and joint hypermobility together in children with ARM. This study aimed to examine trunk muscle endurance, joint hypermobility and postural alignment in children with ARM and to compare these parameters with those of healthy children. We hypothesized that children with ARM would exhibit differences in trunk muscle endurance, joint hypermobility and postural alignment compared with healthy controls.

## 2. Materials and Methods

### 2.1. Study Design and Participants

This study was designed as a case–control research. Ethical approval was obtained from the local ethics committee of the university before the initiation of the study (approval number: 2025/680). The study was carried out between June 2025 and December 2025 in accordance with the principles of the Declaration of Helsinki. Before participation, the children and their parents or legal guardians were informed about the aims and procedures of the study, and written informed consent was obtained from the parents/legal guardians.

The ARM group was recruited using convenience sampling from children with a confirmed diagnosis of ARM who were followed at Bilkent City Hospital and met the predefined eligibility criteria. The control group consisted of age- and sex-matched healthy children recruited from relatives of patients and hospital staff using snowball sampling. ARM diagnosis was based on the clinical diagnosis documented in the patients’ medical records by the pediatric surgeon. ARM subtypes were identified from pediatric surgical records, operative findings, and available preoperative imaging. For descriptive purposes, a study-specific binary grouping was used. Rectourethral prostatic and rectovesical fistulas, together with cloacal malformations, were included in the high-type group, whereas rectoperineal and rectovestibular fistulas, anterior ectopic anus, and anal stenosis were included in the low-type group. The inclusion of cloacal malformations in the high-type group represented a descriptive grouping decision rather than a uniform anatomical designation. The inclusion criteria for the ARM group were being 7–18 years of age, having a diagnosis of ARM, and obtaining written informed consent from parents or legal guardians. The inclusion criteria for the control group were being 7–18 years of age, being healthy, and obtaining written informed consent from a parent or legal guardian. The exclusion criteria in both groups were the presence of a neurological disorder that could affect bowel function, such as spina bifida or cerebral palsy; previous spinal surgery that could alter spinal structure or function; an uncontrolled endocrine or metabolic disorder; and having any other gastrointestinal disease, such as inflammatory bowel disease or celiac disease. A history of lower-extremity surgery and the presence of leg-length discrepancy were assessed during participant evaluation; neither was identified in either group. Neurological, endocrine, and metabolic disorders, as well as constipation and incontinence, were screened based on medical records and medical histories obtained from parents or legal guardians.

### 2.2. Assessments

All evaluations were carried out by face-to-face interviews and physical measurements. Participants’ age (years), body mass index (BMI), sex and medical history, including chronic conditions and surgical history, were assessed. Information regarding urinary and fecal incontinence, constipation, toileting management, and diaper use was also obtained. The assessors were blinded to the participants’ group status during the assessments.

Trunk muscle endurance was evaluated using the McGill Trunk Muscle Endurance Tests, including trunk flexion, trunk extension, and right and left lateral flexion endurance tests. Before each test, participants were asked to complete a practice run. Then, each test was repeated three times, and the best measured score was recorded in seconds (s). The children were positioned according to the standardized procedures for each test and were asked to maintain the required posture for as long as possible [17].

Joint hypermobility was evaluated with the Beighton Hypermobility Score (BHS). The Beighton scoring system evaluates trunk flexion, apposition of the thumb to the forearm, fifth-finger extension, and bilateral elbow and knee hyperextension. Each positive maneuver is assigned 1 point, resulting in a total score ranging from 0 to 9. A total score of 6 or higher was considered indicative of joint hypermobility [18]. Postural alignment analysis was evaluated with the PostureScreen Mobile^®^ application, version 5.2 (PostureCo., Inc., Trinity, FL, USA) [19]. PostureScreen Mobile is an application that measures body alignment, asymmetries, and posture deviations using anterior, lateral, and posterior posture photographs captured with the device’s camera. Before the assessment, participants were instructed to wear tight-fitting clothing to allow clear visualization of body contours and anatomical landmarks. All participants were assessed barefoot. Small markers were placed on the relevant anatomical landmarks to facilitate landmark identification. A total of 36 marker placements were used across four views: 5 in the left lateral view, 5 in the right lateral view, 11 in the anterior view, and 15 in the posterior view. All marker placements and PostureScreen Mobile assessments were performed by the same physiotherapist. During image acquisition, participants stood in a standardized standing position on a marked point on the floor, with their arms relaxed at their sides and their gaze directed forward. The camera was positioned in a standardized manner and maintained perpendicular (90°) to the floor to minimize perspective-related measurement error. The application provides angular and linear measurements of postural alignment. The recorded parameters included head deviation (cm), head tilt angle, shoulder deviation (cm), shoulder tilt angle, hip deviation (cm), and hip tilt angle.

### 2.3. Sample Size and Statistical Analysis

The G*Power (G*Power Ver. 3.1.9.7, Franz Faul, Universität Kiel, Kiel, Germany) package was used for sample size calculation. A pilot study involving the first 15 individuals was conducted before the main study. Based on an effect size of Cohen’s d = 0.723 calculated from the trunk extensor muscle endurance scores obtained in the pilot study, a minimum of 20 participants per group was required to achieve 80% statistical power at an alpha level of 0.05.

The normality of continuous variables included in the study was assessed graphically and using the Shapiro–Wilk test. Descriptive statistics for the variables were presented as mean ± standard deviation (SD) or median (interquartile range [IQR]; 25th–75th percentile), as appropriate. Categorical variables were presented as number (n) and percentage (%), and chi-square (χ^2^) test statistics were reported.

For between-group comparisons, the independent-samples *t*-test was used for normally distributed variables, whereas the Mann–Whitney U test was used for non-normally distributed variables. To evaluate the clinical relevance of the findings, effect sizes were calculated using Cohen’s d for parametric comparisons and the effect size r for non-parametric comparisons. Effect sizes were interpreted according to Cohen’s classification: for r, values of 0.10, 0.30, and 0.50 were considered small, medium, and large effects, respectively; for Cohen’s d, values of 0.20, 0.50, and 0.80 were considered small, medium, and large effects, respectively. Where appropriate, 95% confidence intervals (95% CIs) were calculated for the effect estimates. To account for multiple comparisons, *p* values were adjusted using the Benjamini–Hochberg false discovery rate (FDR) procedure. Statistical analyses and calculations were performed using IBM SPSS Statistics 26.0 (IBM Corp. Released 2019. IBM SPSS Statistics for Windows, Version 26.0. Armonk, NY, USA: IBM Corp.) and MS Excel 2024. The level of statistical significance was accepted as *p* < 0.05.

## 3. Results

**Figure 1:**
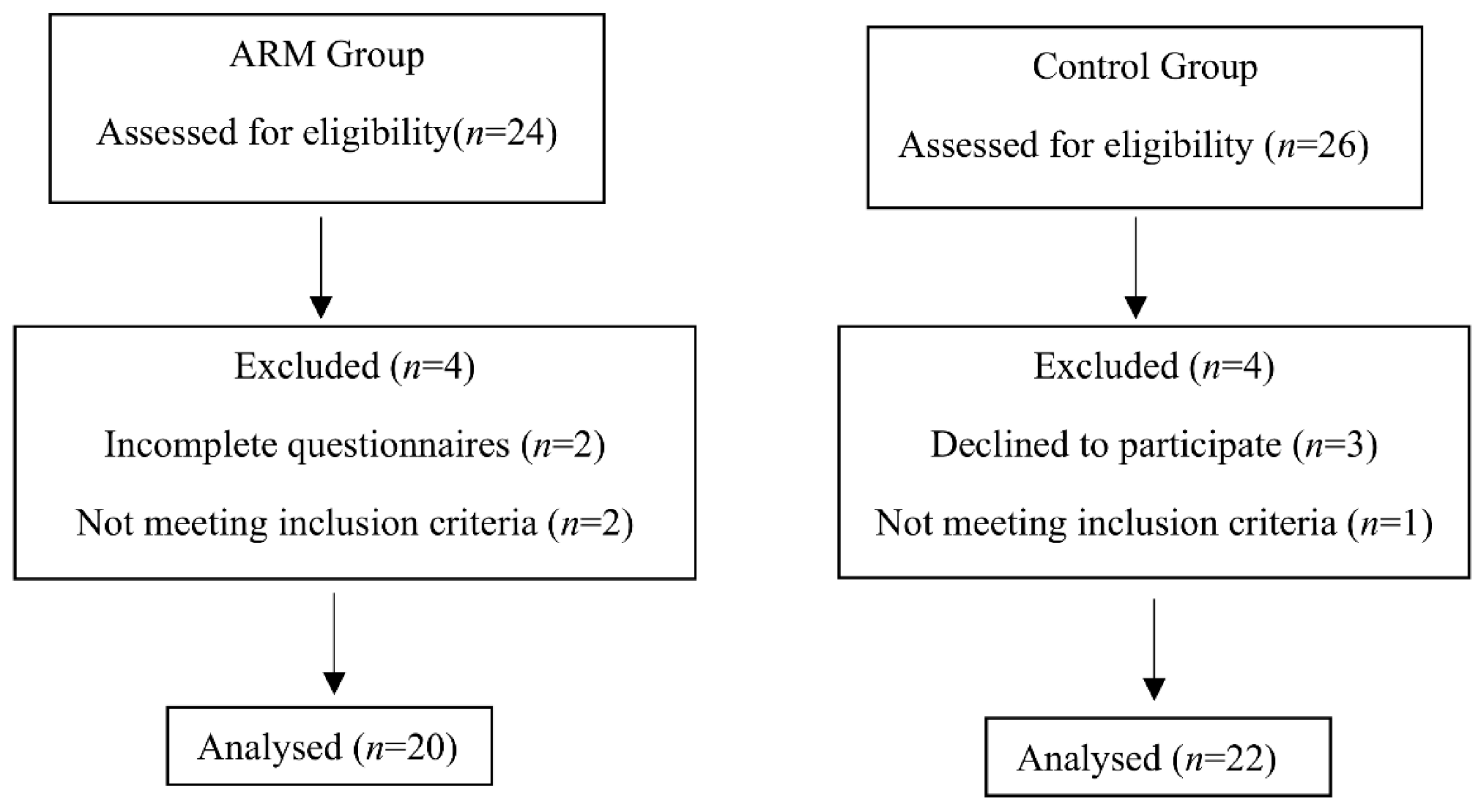
Flowchart diagram for the children.

Fifty children were invited to the study. Forty-two children completed the study (ARM group, *n* = 20; control group, *n* = 22). The flowchart of the participants according to inclusion and exclusion criteria is presented in Figure 1.

### Baseline Characteristics

The groups were similar in terms of age, BMI, sex, and educational level (*p* > 0.05). When the clinical characteristics were examined, the rates of urinary incontinence, fecal incontinence, constipation, catheter use, and diaper use were significantly higher in the ARM group than in the control group (*p* < 0.05). In particular, the ARM group showed notably high rates of fecal incontinence (95%) and constipation (75%). In the ARM group, 55% of the children had high-type ARM and 45% had low-type ARM. (Table 1).

The trunk flexion, extension, right lateral flexion, and left lateral flexion endurance scores were 21.25 ± 9.11 s, 24.0 (14.0–34.5) s, 17.00 ± 11.53 s, and 12.5 (6.0–35.0) s in the ARM group, respectively, whereas the corresponding scores in the control group were 33.64 ± 13.15 s, 30.0 (28.0–35.0) s, 25.64 ± 7.70 s, and 22.0 (20.0–26.0) s, respectively. Trunk flexion, extension, and right lateral flexion endurance were significantly lower in the ARM group, and these differences remained significant after Benjamini–Hochberg FDR correction (FDR *p* = 0.004, 0.043, and 0.013, respectively). Large effect sizes were observed for trunk flexion (d = 1.09; 95% CI: 0.54–1.77) and right lateral flexion endurance (d = 0.89; 95% CI: 0.28–1.71), while extension endurance showed a smaller effect (r = 0.33; 95% CI: 0.02–0.61). Left lateral flexion endurance was not statistically significant after FDR correction (FDR *p* = 0.091) (Table 2).

The BHS was significantly higher in the ARM group than in the control group, and this difference remained statistically significant after FDR correction (FDR *p* = 0.008). The effect size for the BHS difference was moderate (r = 0.41; 95% CI: 0.11–0.69). In addition, hypermobility was observed in 45.0% of the ARM group and 9.1% of the control group, and this difference also remained statistically significant after FDR correction (FDR *p* = 0.008). The odds of hypermobility were approximately 8.2 times higher in the ARM group than in the control group (OR = 8.18; 95% CI: 1.50–44.77) (Table 3).

Postural analysis showed that the right head shift values, right head tilt values, right shoulder shift values, right hip shift values, and left shoulder shift values were significantly greater in the ARM group than in the control group, and these differences remained statistically significant after FDR correction (FDR *p* < 0.001, FDR *p* = 0.013, FDR *p* = 0.013, FDR *p* = 0.002, and FDR *p* = 0.042, respectively). Large effect sizes were observed for the right head shift values (r = 0.61; 95% CI: 0.37–0.81), right head tilt values (d = 0.96; 95% CI: 0.36–1.69), right hip shift values (r = 0.56; 95% CI: 0.31–0.76), and left shoulder shift values (d = 0.80; 95% CI: 0.22–1.45). No statistically significant group differences were observed for the remaining postural variables after FDR correction (FDR *p* > 0.05) (Table 4).

## 4. Discussion

In this study, children with ARM demonstrated reduced trunk muscle endurance and greater joint hypermobility compared with healthy controls. Significant differences were also observed in selected postural alignment parameters, including right head shift, right head tilt, right shoulder shift, right hip shift, and left shoulder shift. In recent years, trunk muscle endurance has increasingly been investigated in individuals with urinary and bowel symptoms [12,20,21]. Karaaslan et al. reported that lumbopelvic muscle endurance, stability, and mobility were lower in children and adolescents with nocturnal enuresis compared with healthy children [12]. Tuncer et al. also reported reduced trunk muscle endurance in children with lower urinary tract dysfunction [20]. Toprak Çelenay et al. reported that trunk muscle flexor, extensor, and right and left lateral flexor endurance, as well as core stability, were lower in individuals with chronic functional constipation compared with healthy controls [21]. To the best of our knowledge, no previous study has examined trunk muscle endurance in children with ARM. In the present study, trunk flexion, extension, and right lateral flexion endurance were significantly lower in children with ARM than in healthy controls. However, no significant difference was observed in left lateral flexion endurance between the groups. Similarly, postural alterations were more prominent on the right side, particularly in the head, shoulder, and hip alignment parameters. The right-sided postural deviations and reduced right-sided trunk muscle endurance observed in children with ARM may be related to the interaction among altered spine posture, frequently observed defecation problems, and the abdominal–pelvic–spine stabilization system. In children with ARM, defecation problems may be associated with repetitive straining and increased intra-abdominal pressure, which could influence abdominal–pelvic floor coordination and trunk stabilization. However, because these symptoms were not assessed using validated instruments, their contribution to the observed trunk and postural findings should be interpreted cautiously. These relationships should therefore be considered hypothetical and require further investigation [11,12].

Joint hypermobility has been discussed in the literature as a factor that may be associated with bladder and bowel dysfunctions in children [14,15,16]. In the present study, the hypermobility score was significantly higher in children with ARM than in healthy controls, and the prevalence of hypermobility was also greater in the ARM group. This result suggests that musculoskeletal factors associated with ARM may contribute to the observed findings [22]. Joint hypermobility was also more common in children with ARM; however, its relationship with postural alterations and trunk muscle endurance was not directly analyzed in the present study. To the best of our knowledge, this is the first study to evaluate generalized joint hypermobility in children with ARM. Therefore, further studies are needed to confirm this finding in different sample groups and to investigate the relationships between hypermobility, posture, trunk muscle endurance and bowel function in children with ARM. In this regard, rehabilitation approaches for children with ARM should not focus solely on bowel symptoms. Assessment of trunk muscle endurance, lumbopelvic stability, and postural alignment may also be important, with these components incorporated into treatment programs when clinically indicated.

The maintenance of healthy spine posture is a dynamic control process achieved through the coordinated interaction among the spine, pelvis, abdominal muscles, and pelvic floor muscles [23]. Abnormalities in spine posture, deviations in postural angles, and changes in the endurance of trunk muscles may be associated with changes in the functional interaction among the abdominal–pelvic–spine stabilization systems [24]. To the best of our knowledge, no previous study has comprehensively evaluated detailed trunk muscle endurance, generalized joint hypermobility and postural alignment in children with ARM. Several studies have been conducted to examine postural alignment, lumbopelvic mobility, trunk muscle endurance, balance, and core muscle properties in children with urinary and fecal symptoms such as nocturnal enuresis, lower urinary tract symptoms, and constipation [22,23,24]. Chase et al. reported that children with slow-transit constipation had reduced trunk control and impaired posture compared with healthy controls [22]. In another study conducted in children with nocturnal enuresis, increased anterior pelvic tilt was demonstrated [23]. Similarly, a study involving children with primary monosymptomatic nocturnal enuresis reported increased lumbar lordosis and sacral–hip angles [24]. In the present study, right head shift, right head tilt angle, right shoulder shift, right hip shift, and left shoulder shift were significantly higher in children with ARM compared with healthy controls. These findings suggest that children with ARM may present with more pronounced asymmetrical postural loading and segmental malalignment, particularly in the frontal plane. The presence of significant differences mainly in shift-related parameters may suggest greater postural asymmetry in the frontal plane rather than a generalized angular postural deviation. Although some shift parameters differed significantly between groups, the clinical relevance of these small 2D differences remains uncertain and should be interpreted cautiously. In the absence of studies directly examining these outcomes in children with ARM, this asymmetric alignment pattern may be interpreted in the context of altered lumbopelvic control and trunk muscle performance reported in related pediatric pelvic floor, bladder, and bowel dysfunctions [12,20,25]. However, since not all postural parameters differed between groups, the findings in the ARM group should be interpreted as selective segmental deviations rather than a global postural impairment. The reason for the predominance of right-sided postural findings remains unclear. Previous studies in other pediatric conditions involving constipation or urinary symptoms have reported postural and lumbopelvic dysfunctions. Although these populations are not directly comparable with children with ARM, our study provides additional evidence by identifying specific head, shoulder, and hip alignment deviations in children with ARM [12,25,26,27,28].

This study has several limitations. First, due to its case–control design, causal relationships among postural alterations, trunk muscle endurance, and hypermobility in children with ARM cannot be established. Second, subgroup analyses according to the type of ARM, surgical procedures, and associated anomalies could not be performed. Third, the relatively small sample size may have limited the statistical power and generalizability of the findings. The recruitment of the control group using snowball sampling from relatives of patients and hospital staff may have introduced some degree of selection bias. In addition, spine structures were not evaluated using radiological imaging, and postural assessment was performed using a two-dimensional mobile application. Therefore, the postural findings should be interpreted cautiously because they may be influenced by measurement and positioning-related factors. PostureScreen Mobile has not been specifically validated in children, and intra-rater/test–retest reliability and measurement error were not evaluated in the present study. Constipation and incontinence were not assessed using validated instruments, which may have introduced measurement or misclassification bias. Potential confounding factors, including physical activity level, sports participation and pain, were not systematically assessed. Future studies with larger sample sizes and multicenter designs are needed to investigate the relationships among posture, trunk muscle endurance, hypermobility, and bowel symptoms in children with ARM in more detail.

## 5. Conclusions

In conclusion, changes in postural alignment, decreased trunk muscle endurance, and increased joint hypermobility were found in children with ARM. These findings suggest that routine assessment of trunk muscle endurance, joint hypermobility, postural alignment and lumbopelvic control may be considered in children with ARM, with individualized rehabilitation provided when clinically indicated.

## Figures and Tables

**Table 1:** Demographic and physical characteristics of the groups.

Parameters	ARM Group (*n* = 20)	Control Group (*n* = 22)	Test Statistic	
			t; z; *χ*^2^	*p*
Age (year, X ± SD)	11.20 ± 2.98	11.04 ± 2.68	t = 0.177	0.861
BMI (kg/m^2^_,_ Median, IQR)	18.25 (15.98–21.16)	17.88 (15.50–19.50)	z = 0.705	0.481
Sex, *n* (%)				
Girl	14 (70.0)	14 (63.6)	$\chi^{2}$ = 0.191	0.662
Boy	6 (30.0)	8 (36.4)		
Education Level, *n* (%)				
Primary	11 (55.0)	10 (45.5)	$\chi^{2}$ = 1.255	0.534
Middle	5 (25.0)	9 (40.9)		
High	4 (20.0)	3 (13.6)		
Fecal Incontinence, *n* (%)				
Yes	19 (95.0)	0 (0.0)	$\chi^{2}$ = 38.165	<0.001 *
No	1 (5.0)	22 (100.0)		
Constipation, *n* (%)				
Yes	15 (75.0)	0 (0.0)	$\chi^{2}$ = 25.667	<0.001 *
No	5 (25.0)	22 (100.0)		

ARM: anorectal malformation, X: mean, SD: standard deviation, *n*: number, %: percentage, *: *p* < 0.05, z: Mann–Whitney U test, t: independent-samples *t*-test,$\;\chi^{2}$: chi-square test, IQR: 25th–75th percentile.

**Table 2:** Comparison of trunk muscle endurance scores of groups.

Parameters	ARM Group (*n* = 20)	Control Group (*n* = 22)	Test Statistic			Effect Size (95% CI)
	Mean ± SD or Median (IQR)	Mean ± SD or Median (IQR)	t or z	*p*	FDR *p*	d, r
Trunk flexor muscle endurance test (s)	21.25 ± 9.11	33.64 ± 13.15	t = 3.514	0.001 *	0.004	1.09 (0.54, 1.77)
Trunk extensor muscle endurance test (s)	24.0 (14.0–34.5)	30.0 (28.0–35.0)	z = 2.143	0.032 *	0.043	0.33 (0.02, 0.61)
Right trunk lateral flexor muscle endurance (s)	17.00 ± 11.53	25.64 ± 7.70	t = 2.879	0.006 *	0.013	0.89 (0.28, 1.71)
Left trunk lateral flexor muscle endurance test (s)	12.5 (6.0–35.0)	22.0 (20.0–26.0)	z = 1.693	0.091	0.091	0.26 (−0.58, 0.09)

*: *p* < 0.05, t: independent-samples *t*-test, SD: standard deviation, IQR: 25th–75th percentile, s: second, FDR *p*: *p* value adjusted for multiple comparisons using the Benjamini–Hochberg procedure.

**Table 3:** Comparison of hypermobility values of the groups.

Parameters	ARM Group (*n* = 20)	Control Group (*n* = 22)	Test Statistic			Effect Size (95% CI)
			z; *χ*^2^	FDR *p*	*p*	
BHS, Median (IQR)	5.0 (3–6)	2.5 (1–3)	z = 2.662	0.008	0.008 *	r = 0.41 (0.11, 0.69)
Hypermobility, *n* (%)						
Yes	9 (45.0)	2 (9.1)	χ^2^ = 6.988	0.008	0.008 *	OR: 8.18 (1.50, 44.77)
No	11 (55.0)	20 (90.9)				

ARM: anorectal malformation, BHS: Beighton Hypermobility Score, IQR: 25th–75th percentile, z: Mann–Whitney U test, *: *p* < 0.05, *n*: number, %: percentage, *χ*^2^: chi-square test, FDR *p*: *p* value adjusted for multiple comparisons using the Benjamini–Hochberg procedure, OR: odds ratio. Effect size was reported as r for the BHS and as OR for the presence of hypermobility.

**Table 4:** Comparison of posture analysis values of the groups.

Parameters	ARM Group (*n* = 20)	Control Group (*n* = 22)	Test Statistic			Effect Size (95% CI)
	Mean ± SD or Median (IQR)	Mean ± SD or Median (IQR)	z; t	*p*	FDR *p*	d, r
Right Head Shift (cm)	2.56 (1.93–4.01)	0.85 (0.62–1.18)	z = 3.954	<0.001 *	<0.001	0.61 (0.37, 0.81)
Right Head Tilt Angle (°)	16.38 ± 8.53	9.75 ± 5.07	t = 3.093	0.004 *	0.013	0.96 (0.36, 1.69
Right Shoulder Shift (cm)	1.45 (0.73–2.10)	0.51 (0.35–1.27)	z = 2.860	0.004 *	0.013	0.44 (0.17, 0.68)
Right Shoulder Tilt Angle (°)	3.28 (0.0–4.42)	2.22 (0.0–4.35)	z = 0.796	0.426	0.512	0.12 (−0.18, 0.43)
Right Hip Shift (cm)	1.25 (0.69–1.77)	0.35 (0.17–0.64)	z = 3.603	<0.001 *	0.002	0.56 (0.31, 0.76)
Right Hip Tilt Angle (°)	2.46 (1.0–4.12)	0.0 (0.0–3.76)	z = 1.264	0.206	0.309	0.20 (−0.11, 0.49)
Left Head Shift (cm)	1.14 (0.71–1.81)	0.74 (0.60–1.23)	z = 1.411	0.158	0.271	0.22 (−0.09, 0.52)
Left Head Tilt Angle (°)	6.73 (4.18–14.01)	7.36 (5.55–14.13)	z = 0.416	0.678	0.678	0.06 (−0.36, 0.24)
Left Shoulder Shift (cm)	1.47 ± 1.13	0.75 ± 0.65	t = 2.512	0.018 *	0.042	0.80 (0.22, 1.45)
Left Shoulder Tilt Angle (°)	2.24 (0.0–4.48)	1.08 (0.0–4.00)	z = 0.714	0.475	0.519	0.11 (−0.19, 0,41)
Left Hip Shift (cm)	0.78 (0.41–1.35)	0.60 (0.32–1.04)	z = 1.197	0.231	0.309	0.18 (−0.12, 0.47)
Left Hip Tilt Angle (°)	2.21 (0.0–3.52)	3.61 (2.30–4.83)	z = 1.669	0.095	0.190	0.26 (−0.54, 0.04)

ARM: anorectal malformation, *: *p* < 0.05, z: Mann–Whitney U test, t: independent-samples *t*-test, IQR: interquartile range, SD: standard deviation, IQR: 25th–75th percentile, d: Cohen’s d effect size, r: effect size for Mann–Whitney U test, FDR *p*: *p* value adjusted for multiple comparisons using the Benjamini–Hochberg procedure. Shift values are expressed in cm and tilt angles in degrees (°).

## Data Availability

Data generated and analyzed in this study can be obtained from the corresponding author upon reasonable request, as access is limited by ethical approval requirements.
